# Prevalence of Emergence Delirium in Children Undergoing Tonsillectomy and Adenoidectomy

**DOI:** 10.1155/2022/1465999

**Published:** 2022-09-28

**Authors:** Katie Liu, Christopher Liu, Seckin O. Ulualp

**Affiliations:** ^1^Department of Anesthesiology and Pain Management, Division of Pediatric Anesthesiology, University of Texas Southwestern Medical Center, Dallas, TX, USA; ^2^Department of Otolaryngology-Head and Neck Surgery, Division of Pediatric Otolaryngology, University of Texas Southwestern Medical Center, Dallas, TX, USA

## Abstract

**Objective:**

Emergence delirium (ED) is associated with behavioral disturbances and psychomotor agitation, increased risk of selfinjury, delayed discharge, and parental dissatisfaction with quality of care. Otolaryngology procedures are associated with an increased risk of ED. The aims of this study were to determine the prevalence of ED in children who had tonsillectomy and adenoidectomy (T&A), assess the characteristics of children who had ED, and ascertain the recovery times of patients with ED.

**Methods:**

Charts of patients who had tonsillectomy and adenoidectomy between Jan 1, 2018 and March 26, 2020 at a tertiary children's hospital were reviewed. Data collection included demographics, body mass index, indication for T&A, Pediatric Anesthesia Emergence Delirium (PAED) score, American Society of Anesthesiologists (ASA) physical status classification, total anesthesia time, postanesthesia care phase I time, and postanesthesia care phase II time.

**Results:**

Of the 4974 patients who underwent T&A, ED occurred in 1.3% of patients. Toddlers (2.9%) and male children (1.6%) had a significantly higher prevalence of ED. Prevalence of ED was similar amongst patients with recurrent tonsillitis, patients with obstructive sleep disordered breathing, and patients with both obstructive sleep apnea (OSA) and recurrent tonsillitis. The prevalence of ED was not different amongst ASA I, ASA II, and ASA III. Males with ED had longer total anesthesia times (41 *v*. 34 minutes, *p*=0.02) and ASA I patients with ED had longer phase I times (*p*=0.04) in the postanesthesia care unit (PACU). There was no significant difference in total anesthesia time, phase I time, or phase II time when compared across the subgroups of gender, age, indication for T&A, severity of obstructive sleep apnea (OSA), and ASA score.

**Conclusions:**

Males, toddlers, and preschool-age children were more likely to have ED. Males with ED had longer total anesthesia times. ED was associated with longer phase I times in ASA I patients.

## 1. Introduction

Emergence delirium (ED) is a frequent complication in children undergoing general anesthesia, with an incidence ranging from 10% to 80% depending on the criteria used to define it [1–6]. ED is a complex of behavioral disturbances and psychomotor agitation associated with an altered state of consciousness during emergence from anesthesia [7]. ED is usually brief, but the disorientation and agitated behavior associated with ED can increase the risk of injury to self or others, require greater postoperative nursing resources, delay discharge, and decrease the patient and caregiver satisfaction with the care experience [1, 5, 7–9]. Age, preoperative anxiety level, preoperative behavior, volatile anesthetics, and type of surgery have been suggested as risk factors for ED in children [1, 6, 10–21].

Otolaryngology procedures have been suggested as a risk factor for ED [1, 5]; the pathogenesis and prevalence of ED in children undergoing otolaryngologic procedures have not been identified. Tonsillectomy is the second most common ambulatory procedure performed on children in the US. [22]. It is commonly performed to treat obstructive sleep disordered breathing, obstructive sleep apnea (OSA), and recurrent tonsillitis. Inadequate pain control and ED may interfere with recovery after T&A. Better understanding of the characteristics of ED in children undergoing T&A may improve the quality of anesthetic and otolaryngologic care in this patient population.

Our primary objective was to determine the prevalence of ED in children who had T&A at our institution and assess the characteristics of children who had ED. Our secondary objective was to assess the recovery times of patients with ED to determine the effects of ED on the postoperative care experience.

## 2. Materials and Methods

The charts of patients who had undergone T&A between January 1, 2018 and March 26, 2020 were retrospectively reviewed to identify children who developed ED. The local institutional human research review board approved the study. Patients who had recurrent tonsillitis, obstructive sleep disordered breathing or OSA, or both were included in the study. Exclusion criteria were lack of Pediatric Anesthesia Emergence Delirium (PAED) scale in the patient record [23] and procedures involving T&A in combination with a nonotolaryngologic procedure.

### 2.1. Anesthesia Protocol

The anesthesia protocol consisted of preoperative administration of midazolam either orally or intravenously. If a preoperative intravenous (IV) catheter was not placed, mask induction proceeded with sevoflurane and nitrous oxide in oxygen followed by placement of a peripheral IV cannula. After peripheral IV cannula insertion, propofol and fentanyl were administered for induction. In some cases, a neuromuscular blocking agent such as rocuronium or succinylcholine was administered to facilitate endotracheal intubation. After intubation, general anesthesia was maintained with sevoflurane in oxygen and air or nitrous oxide with controlled ventilation. All children received acetaminophen, dexamethasone, and ondansetron. If a nondepolarizing neuromuscular blocking agent was utilized, blockade was reversed using either neostigmine or sugammadex at the end of the procedure. All patients were transferred to the postanesthesia care unit (PACU) after removal of the endotracheal tube. Postoperative pain was assessed every 15 minutes using the Face, Legs, Activity, Cry, Consolability (FLACC) or FACES pain scales depending on patient age, developmental level, and nursing discretion [24–27]. Pain was managed with ibuprofen with or without opioids at the discretion of the anesthesiologist. PAED score was assessed by the recovery room nurse assigned to care for the patient [23]. The PAED score was calculated upon patient awakening and prior to medication administration in order to differentiate pain from ED. A total PAED score equal to or greater than 10 in PACU indicated emergence delirium [23].

### 2.2. Clinical Assessment

The indications for T&A were recurrent tonsillitis, obstructive sleep-disordered breathing or OSA, or both. The American Academy of Otolaryngology Head and Neck Surgery guidelines were used to determine indications for surgery [28]. Polysomnography (PSG) was performed in a sleep laboratory of a tertiary care children's hospital. The criteria of the American Academy of Sleep Medicine (AASM) were used to determine sleep measurements [29]. All patients with a diagnosis of OSA had a preoperative polysomnography study; no children were excluded based on PSG results.

### 2.3. Data Analysis

Data collection from charts included age, gender, obesity, comorbid conditions, American Society of Anesthesiologists (ASA) physical status classification, indication for T&A, OSA severity, intraoperative use of fentanyl or dexmedetomidine, PAED score, total anesthesia time (from the time of anesthesia start to the end of anesthesia care), postanesthesia care phase I time (the time to recover from anesthesia and return to baseline vital signs), and postanesthesia care phase II time (the time from phase I to transfer to ward or home). Patients were categorized into the following age groups: toddler (1-3 years), preschooler (3-5 years), middle childhood (6-11 years), and teenager (12-18 years) [30].

The prevalence of ED was assessed in the entire group of patients. The effects of age, gender, ASA status, anesthesia time, postanesthesia care phase I time, and postanesthesia care phase II time on PAED score were evaluated in children with ED. Statistical comparisons between groups were performed using parametric (one‐way analysis of variance) or nonparametric test (Kruskal–Wallis one‐way analysis of variance on ranks). Comparisons of rate of ED were performed by a *χ*2 test or Fisher exact test, as appropriate. A *p* value of <0.05 was considered significant. Data are presented as mean ± standard deviation.

## 3. Results

### 3.1. Prevalence of Emergence Delirium

Four thousand nine hundred seventy-four children (age range: 1-18 years, 7.0 ± 3.7) had undergone T&A (Table 1). The most common indication for surgery was obstructive sleep disordered breathing (79.2%) (Table 1). ED occurred in 64 children (age range: 2-18 years, 5 ± 3). The prevalence of ED was 1.3%. Boys had a higher prevalence of ED than girls (*p*=0.03) (Table 1). Toddlers had a higher prevalence of ED than preschooler (*p*=0.04), middle age (*p* < 0.001), and teenage children (*p*=0.01). Preschoolers had a higher prevalence of ED than middle age children (*p*=0.02). The prevalence of ED was similar between middle age and teenage children (*p*=0.9) as well as between preschooler and teenage children (*p*=0.2). Prevalence of ED was similar amongst patients with recurrent tonsillitis (1.8%), patients with obstructive sleep disordered breathing (1.3%), and patients with both OSA and recurrent tonsillitis (0.9%) (*p*=0.9). The prevalence of ED was not different amongst ASA I (1.4%), ASA II (1.2%), and ASA III (1.5%) (*p*=0.6).

### 3.2. Characteristics of Children with Emergence Delirium

In children with ED, comorbid conditions included asthma in 14 children, repaired congenital heart abnormality in 3, developmental delay in 3, autism in 3, and seizure in 1. Forty-nine children were nonobese and 15 were obese (Table 2). The majority of children with ED (90%) received intraoperative dexmedetomidine. Intraoperative fentanyl was administered in 73% of the children with ED. Total anesthesia time ranged from 21 to 106 minutes (41 ± 13, median = 40). Total phase I time ranged from 20 to 323 minutes (100 ± 58, median = 81). PAED score ranged from 10 to 20 (15 ± 3, median = 16). Total anesthesia time, total phase I time, total phase II time, and PAED scores in children with ED were similar among all the studied subgroups of age, gender, indication for T&A, severity of OSA, ASA score, and administration of intraoperative dexmedetomidine and fentanyl (Table 2) (*p* > 0.05). The exceptions included significantly longer total anesthesia time in males (*p*=0.02) and phase I time in ASA I patients (*p*=0.004) (Table 2).

Total anesthesia time was not different between children with ED (40 ± 11, median = 41) and children without ED (38 ± 10, median = 35) (*p*=0.2). Total phase I time in children with ED (100 ± 58, median = 81 minutes) was longer than that of children without ED (70 ± 46, median = 58 minutes) (*p* < 0.001).

## 4. Discussion

In the present study, the prevalence of ED was 1.3% in children undergoing T&A. ED is a very common PACU occurrence in children undergoing general anesthesia with an overall incidence of 10% to 80% in children undergoing anesthesia for a variety of procedures [1–7, 23, 31, 32]. The rate of ED ranges from 13% to 26% in children undergoing otolaryngologic procedures [5, 33]. Earlier studies have reported that ED is associated with various patient and procedural risk factors, including younger age, volatile anesthetic use, and type of surgery including otorhinolaryngology procedures [1, 5, 34–36]. Other risk factors for ED identified in the literature include pain, patient and parental anxiety, pre-existing behaviors, and patient/parent interaction with healthcare providers [32, 34]. Plausible explanations of lower rate of prevalence of ED in our study are differences in demographics, preoperative level of anxiety, anesthetic agent, and postoperative pain control. Lower incidences of ED reporting by PACU nurses at our institution could also contribute to the lower rate of ED in our study.

The rate of ED in varying age groups of children undergoing anesthesia has not been systematically studied. Some studies suggest that ED occurs more frequently in children ages 2-5, but the mechanism behind this finding is unclear [37–39]. At the time the PAED scale was developed, PAED score was negatively correlated with age in a group of children ASA I or II and aged 6 to 18 years [23]. In previous studies published, prior to the development of PAED scale, emergence agitation occurred more often in younger children. Incidence of emergence agitation was 13% to 18% in children between 3-9 years of age and 9% in children between 10-19 years [1, 10]. We assessed the rate of ED in different developmental age groups. Our findings of increased rates of ED in younger children (toddler and preschool) are consistent with the current literature [1, 10]. The prevalence of ED was similar between middle age and teenage children. The effect of age on the incidence of ED appears to dissipate after preschool years.

Our finding of male gender as a risk factor for ED is also consistent with what has been reported [40]. We also found that males with ED had longer total anesthesia time in the recovery room and ASA I patients had longer phase I times. In addition, the association between ED and ASA status as well as indication for T&A has not been previously examined. In this study, the rate of ED was not significantly different when comparing ASA status and indication for T&A.

Having identified higher rates of ED in toddler and preschool-age children, anesthesiologists and otolaryngologists may utilize the present study findings to develop pre- and postanesthetic management as well as counseling strategies to decrease the incidence and impact of ED in children undergoing T&A. An understanding of the risk factors for ED in children undergoing T&A is necessary to prevent, rapidly identify, and treat ED. The limitations of the present study are inherent to the retrospective study design. The first limitation is the lack of information regarding patient-related confounding factors such as pre-existing behaviors of children, level of anxiety prior to surgery, and quality of patient interaction with healthcare providers. While we cannot assess the effect of these confounding factors on our findings, the rate of ED in this group of children was still lower than the previous reports. Another limitation of our study is the use of varying adjuvant anesthetic agents. As the etiology of ED is not yet fully understood, prevention and treatment of ED are variable and inconsistent. Different pharmacologic agents including dexmedetomidine, fentanyl, and propofol are utilized to reduce ED. Use of these agents was not found to be significantly associated with rates of ED, but further studies are needed to better understand the relationship between the use of these agents and ED. Given the known ED risk factors including age, gender, and preoperative anxiety, it is possible that certain agents such as dexmedetomidine were used pre-emptively to help reduce the possible occurrence of ED. The assessment of ED as well as the PAED scale is a subjective clinical determination; therefore, we cannot eliminate potentially inconsistent documentation of PAED score across all care providers.

Present study findings illustrate the need to create a system to uniformly track and evaluate ED, accurately document PAED scores to more fully capture all cases of ED in the PACU, determine strategies to identify which children are at risk for ED, and develop methods to reduce the incidence of ED. Prospective trials assessing the prevalence and risk factors of ED in children undergoing T&A could improve the management of and potentially prevent ED. Prospective trials assessing various interventions in the management of ED and their effect on PACU recovery times would also have the potential of improving our management of ED and impacting overall costs to the patient and the hospital.

## 5. Conclusion

The prevalence of ED was 1.3% in children undergoing T&A in our institution. Males, preschool- and toddler-age children were more likely to have ED. Male patients with ED had significantly longer total anesthesia time, and ASA I patients had significantly longer phase I time in the PACU. Future prospective studies in children undergoing T&A are needed to decrease the incidence of ED, identify the additional risk factors for ED, enhance pre-and postoperative anesthetic management, and improve counseling of caregivers.

## Figures and Tables

**Table 1:** Characteristics of study patients.

Patient group	Number of subjects	Prevalence of emergence delirium	*p*-Value
Entire cohort	4974	1.3%	
Gender			
Male	2658	1.6%	*p*=0.03
Female	2316	0.9%	

Age group			
Toddler	568	2.9%	^*∗*^ *p* < 0.05
Preschooler	1761	1.5%	^†^ *p*=0.02
Middle childhood	2045	0.7%	
Teenager	600	0.8%	

Diagnosis			
Recurrent tonsillitis/pharyngitis	273	1.8%	*p*=0.6
Obstructive sleep disordered breathing (SDB)	3940	1.3%	
Recurrent tonsillitis/pharyngitis + SDB	761	0.9%	

ASA status			
I	210	1.4%	*p*=0.9
II	3658	1.2%	
III	1102	1.5%	
IV	4	0	

^*∗*^Toddlers had higher prevalence of PAED than preschooler, middle childhood, and teenage children. ^†^Preschoolers had higher prevalence of PAED than middle childhood group.

**Table 2:** Comparison of studied variables in children with emergence delirium.

Group		PAED score	Total Anesthesia Time	Total Phase I Time	Total Phase II time
Gender	Male (*n = *43)	15 + 3	43 + 13^*∗*^	102 + 51	120 + 59
	Female (*n = *21)	15 + 3	35 + 10	95 + 72	129 + 39

Age	Toddler (*n = *17)	14 + 3	40 + 8	100 + 54	153 + 4
	Preschooler (*n = *28)	15 + 3	42 + 16	98 + 51	112 + 46
	Middle childhood (*n = *14)	14 + 3	37 + 7	101 + 69	123 + 40
	Teenager (*n = *5)	15 + 3	44 + 19	116 + 101	123 + 50

Obesity	Obese (*n = *15)	15 + 3	44 + 21	125 + 88	112 + 65
	Nonobese (*n = *49)	15 + 3	40 + 9	92 + 44	126 + 50

Comorbidities	Yes (*n = *29)	16 + 2	43 + 15	111 + 64	139 + 73
	No (*n = *35)	14 + 3	40 + 10	93 + 54	118 + 40

ASA Status	I (*n = *3)	15 + 4	41 + 14	83 + 36^†^	91 + 25
	II (*n = *44)	14 + 3	40 + 10	85 + 45	128 + 53
	III (*n = *17)	16 + 3	44 + 18	142 + 72	125 + 80

Indication for surgery	Recurrent tonsillitis/pharyngitis (*n = *5)	14 + 3	43 + 15	94 + 71	127 + 67
	Obstructive SDB (*n = *52)	14 + 3	39 + 9	103 + 49	126 + 26
	Recurrent tonsillitis/pharyngitis + SDB (*n = *7)	16 + 3	41 + 14	99 + 63	118 + 36

OSA severity	Mild OSA (*n = *9)	15 + 2	387 + 8	132 + 102	150 + 12
	Moderate OSA (*n = *6)	18 + 3	42 + 5	7 + 23	129 + 17
	Severe OSA (*n = *7)	16 + 3	55 + 29	116 + 67	-

Intraoperative dexmedetomidine	Yes (*n = *58)	15 + 3	41 + 13	103 + 59	126 + 53
	No (*n = *6)	17 + 3	38 + 10	69 + 33	113 + 43

Intraoperative fentanyl	Yes (*n = *47)	15 + 3	81 + 14	94 + 14	127 + 54
	No (*n = *17)	16 + 3	40 + 8	102 + 62	119 + 49

^*∗*^ *p*=0.02; ^†^*p*=0.004

## Data Availability

Data are available on request.
